# The impact of environmental factors during maternal separation on the behaviors of adolescent C57BL/6 mice

**DOI:** 10.3389/fnmol.2023.1147951

**Published:** 2023-05-24

**Authors:** Sangyep Shin, Sukwon Lee

**Affiliations:** Developmental Disorders and Rare Disease Research Group, Korea Brain Research Institute (KBRI), Daegu, Republic of Korea

**Keywords:** maternal separation (MS), environmental factor, adolescence, early-life stress, C57 (C57BL/6J)

## Abstract

Neonatal maternal separation is a widely used method to construct an early-life stress model in rodents. In this method, pups are separated from their mothers for several hours every day during the first 2 weeks of life, which results in adverse early-life events. It is a known fact that maternal separation can exert a significant impact on the behavior and psychological health, such as anxiety and depression, in adolescent offspring. However, environmental conditions during maternal separation can differ such as the presence of other animals or by placing pups in a different dam. To investigate the differential effects of various conditions of maternal separation on the behavior of adolescent mice, we created the following groups: (1) iMS group: pups were moved to an isolated room with no other adult mice in a nearby cage, (2) eDam group: the pups randomly exchanged their dams, (3) OF group: pups were shifted to another cage with the bedding material containing maternal odor (olfactory stimulation), and (4) MS group: pups were shifted to another vivarium. From postnatal day (PND) 2–20 (i.e., 19 consecutive days), pups were separated from the dam daily for 4 h and exposed to various environments (MS, iMS, eDam, and OF) or were left undisturbed [control (CON) group]. A series of behavioral assessments were conducted to evaluate locomotion, anxiety, recognition, learning, and memory in adolescent offspring. The results showed that neonatal maternal separation led to impaired recognition memory, motor coordination, and motor skill learning across all groups. However, the iMS group exhibited anxiety-like behavior in the elevated plus maze test and enhanced the extinction of fear memory in the auditory fear conditioning test. The OF and eDam groups displayed partially recovered short-term working memory in the Y-maze test but exhibited opposite exploratory behaviors. The OF group spent more time in the center, while the eDam group spent less time. These findings demonstrated that exposure to different environmental conditions during maternal separation causes behavioral alterations in adolescent offspring, providing a potential explanation for the variation in behavioral phenotypes observed in the early-life stress models.

## Introduction

Environmental enrichment has been shown to exert positive effects on stress in both acute and chronic situations, with various studies reporting lower levels of plasma corticosterone (Meijer et al., 2007), reduced anxiety-like behavior (Benaroya-Milshtein et al., 2004), and improved cognitive function (Pham et al., 1999). Conversely, environmental stressors have been shown to increase susceptibility to future stress, resulting in anxiety and depression-like behavior in animals subjected to chronic social defeat stress (Pena et al., 2019). The neurological effects of enrichment were indicated by the changes in the morphology of occipital cortical neurons (Walsh, 1981) and synaptic connections (Globus et al., 1973). Given the importance of environmental factors in animal welfare and their influence on behavioral outcomes (Bayne, 2018), studying their effects on early-life stress models could provide valuable guidance for developing appropriate stress models in the future.

Since its introduction in the 1960s, the concept of maternal separation has been extensively studied in various fields. In addition, several models have been developed to assess the impact of early-life stress on behavioral changes associated with stress response and emotional disorders (Weininger, 1954; Denenberg, 1962; Levine, 1967; Shin et al., 2016). In spite of the detrimental effects of maternal separation, some studies have reported contradictory outcomes (Tsuda and Ogawa, 2012; Akillioglu et al., 2015; Bian et al., 2015; Shin et al., 2016). Maternal separation has been linked to increased susceptibility to anxiety and depression in animal models (Kalinichev et al., 2002; Romeo et al., 2003; Parfitt et al., 2004; Murgatroyd et al., 2009), while other studies have found no difference or decreased anxiety- and depression-like behavior in the experimental animals when compared to that observed in the control mice (Millstein and Holmes, 2007; Parfitt et al., 2007; Gapp et al., 2014; Pierce et al., 2014; Shin et al., 2016; Zoicas and Neumann, 2016). These inconsistencies have been attributed to various parameters, such as species, sex, stressors, and their relevance to animal development (Andersen and Teicher, 2009; Murgatroyd and Spengler, 2011; Tractenberg et al., 2016), which have been shown to affect the behavioral phenotypes of the mice. For instance, male mice that were separated from their mothers exhibited anxiety and fearful behaviors in adulthood, while female mice produced from the same litter showed a reduction in anxiety and fear response during the diestrous phase (Romeo et al., 2003). In another study, the effects of maternal separation on the behavior of C7BL/6 and Balb/c mice were compared, which demonstrated strain-specific and sex-dependent effects during adolescence (Kundakovic et al., 2013). Moreover, the duration of maternal separation has been shown to produce different behavioral phenotypes and also determine the presence of long-term effects (Banqueri et al., 2017). Therefore, a standard model has not been established, and hence, extensive research is required to address these discrepancies.

Various discrepancies have been frequently observed in stress models, which can make it challenging to reproduce the experimental results and disrupt steady research progress on maternal separation. While attempts have been made to classify methodological variations in the experimental protocols of maternal separation (Tractenberg et al., 2016), investigating the issue in terms of sensory modalities (olfactory, auditory, and tactile) has not yet been done. Studies involved in conducting behavioral experiments in adolescent C57BL/6 mice have shown that anxiety-like behaviors were observed only when maternal separation was performed in isolated rooms (Kundakovic et al., 2013; Shin et al., 2016), while separation with cross-fostering, where communication with other dams was allowed, did not induce anxiety-like behavior (Luchetti et al., 2015). Therefore, we imposed additional manipulations in the maternal separation procedure to determine whether the environmental factors could influence the behavioral phenotype of adolescent offspring. Pups were moved to the other vivarium (MS group) or they were moved to an isolated room to block sensory stimulation from other mice (iMS group) or they were shifted to a new cage containing the mother's feces to induce olfactory stimulation (OF group) or dams were randomly assigned to the pups during separation to provide tactile stimulation (eDam group). As a result, an increase in anxiety-related behavior and extinction memory were observed in the iMS group, and spatial working memory was partially recovered in the OF and eDam groups. However, pups in the eDam group showed aggravated exploratory behaviors. These results demonstrate that exposure to various environmental factors during maternal separation cause behavioral alternations in adolescent offspring. Our findings suggest that the use of different environmental factors during maternal separation could explain the inconsistent results of previous studies and, hence, emphasize the need to consider contextual features during the construction of stress models.

## Materials and methods

###  Experimental subjects and behavioral assessment

Pregnant female C57BL/6 mice were obtained from a commercial supplier (KOATECH, Gyeonggi-do, Korea) and transported to the facility at least 1 week before parturition to acclimatize them to the surrounding environment. Litters born before 10:00 a.m. each day were selected, and the day of birth was designated as postnatal day 0 (PND 0). Later, the pups were randomly allocated to each experimental group (CON, MS, iMS, eDam, or OF). To avoid experimenter-induced behavioral variance in mice, a single researcher performed all the experimental tasks during the study. Since all groups were not tested at the same time, each experimental group was compared with the control group, and all behavioral assessments were performed with a corresponding control group. Each group was tested only once in a series of behavioral assessment tasks, and behavioral tests were performed in the following order to minimize the potential stress caused by the task: open-field test, Y-maze test, novel object recognition test, object-location memory test, social interaction test, rotarod test, and auditory fear conditioning test. Mouse behavior was recorded using EthoVision software (Noldus, Leesburg, VA, USA), and the data were used for further offline analysis. All experiments were conducted during the light cycle. All mice were maintained on a 12-h light/dark schedule (lights on from 08:00 a.m. to 08:00 p.m.), and food and water were provided *ad libitum*. Temperature was maintained at 21 ± 2°C throughout the year. All experiments were approved by the Institutional Animal Care and Use Committee of the Korea Brain Research Institute (KBRI).

### Maternal separation procedure

**Figure 1:**
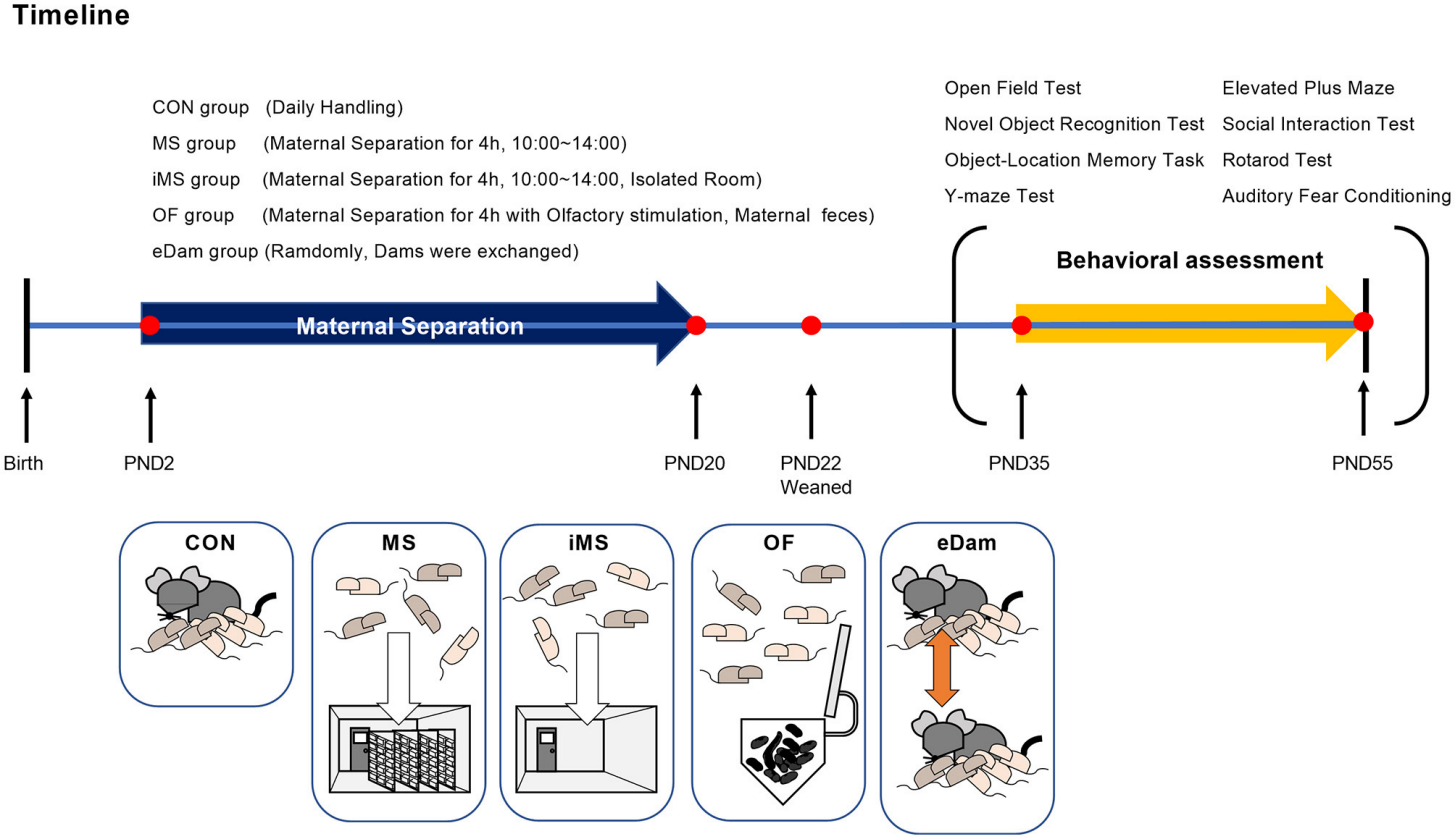
Timeline of maternal separation and experimental groups. CON group, brief handling during PND 2–20; MS group, maternal separation during PND 2–20; iMS group, maternal separation conducted in an empty room during PND 2–20; OF group, maternal separation with their dam's feces during PND 2–20; eDam group, random exchange of dams during PND 2–20. All mice were weaned and housed in cages on PND 22. Five groups were used for each series of behavioral tests in the order of light stress during the adolescent period (PND 35–55).

The basic procedure of maternal separation was performed as described in a previous study (Shin et al., 2016, 2019). Since additional environmental factors were used in this study, a total of four experimental groups were created: MS, iMS, OF, and eDam. To obtain pregnant mice in the same stage of pregnancy, we purchased the mice from a vendor rather than breeding them in a facility and then conducted maternal separation after birth. All pregnant mice had no previous history of delivery, were of the same age at adulthood (13 weeks), and were brought to our vivarium 1 week before delivery. After birth, all the litter groups, except the control group, were separated from their dams for 4 h every day, and this step was followed consecutively from PND 2 to PND 20. To safely separate the litters from dams, dams were first moved to a clean cage, and then, the investigator grasped the bedding in the home cage to minimize the influence of the experimenter's scent. Afterward, the litters were carefully transferred to a new cage with a lid. Dams were returned to the home cage and left undisturbed until the litters were placed back in the home cage. The litters underwent maternal separation according to the experimental groups allocated to them. The litters assigned to the MS group were moved to another vivarium during separation to exclude olfactory or ultrasonic vocalizations between dams and litters. The litters assigned to the isolated MS (iMS) group were placed in an isolated room with no other mice present in a nearby cage. The litters assigned to the olfactory stimulation (OF) group were separated from dams for 4 h, similar to the litters of the MS group, but a small tube containing maternal feces and bedding was placed along with the pups. The litters assigned to the exchange-of-the-dam (eDam) group were randomly exchanged in their dams for 4 h. The litters included in the control group were briefly transferred to the new cage and immediately returned to the home cage for handling purposes but were not separated from their dams. The litters were returned to their home cages after the separation period was over in a manner similar to that followed for the separation process. On PND 22, all litters were separated based on sex and treatment conditions and were maintained in group-housing systems. Only male subjects underwent behavioral assessments during adolescence (PND 35–55). The timeline of the experiment is shown in Figure 1.

### Open-field test

Adolescent offspring were placed in the center of an arena (40 × 40 cm^2^) and allowed to explore the field for 30 min. The recorded video files were used to analyze four parameters: distance moved (total distance traveled throughout the test), velocity (average speed of movements), time spent in the corners (10 × 10 cm^2^, square), and time spent in the center (20 × 20 cm^2^, square).

### Elevated plus maze test

Mice were tested using an elevated plus maze for 10 min. The apparatus consisted of two open arms (30 × 30 cm^2^) and two closed arms (30 × 30 cm^2^) that were positioned 50 cm above the floor. Each mouse was placed in the central platform facing one of the open arms, and the duration spent in the arms was determined. The percentage of time spent in the open (or closed) arms was calculated as the duration in open arms (or closed arms)/total exploring duration (600 s).

### Y-maze test

The experimental mouse was placed at the center of three arms (30 cm) and allowed to freely explore the Y-shaped maze. The total number of visits and total alternations were calculated using EthoVision 16XT software. Entry into an arm was defined as the animal placing all four paws in a particular arm, and alternation was defined as visits to all three arms on consecutive occasions. The alternation index (%) was calculated as follows: the number of alternations divided by the total number of visits minus 2 (maximum alternations) and then multiplying the result by 100.

### Novel object recognition test

The novel object recognition test was conducted in two sessions (familiarization and test sessions). In the familiarization session, mice were allowed to explore the open field arena (40 × 40 cm^2^) containing two identical objects for 10 min. The objects (Lego bricks or Falcon tissue culture flasks filled with bedding) were placed in a corner 6 cm away from the walls and were located on one side of the wall. To prevent the animals from climbing, the height of the objects was >15 cm. After 4 h, one of the objects was replaced with a novel one, and the mice were allowed to explore the arena again for 10 min. The time spent by the mouse with the new object was calculated using EthoVision 16XT software equipped with a deep learning-based tracking algorithm. The software tracked the nose-point of the mouse and calculated the time spent on the object's outer contour (2 cm). The discrimination index was calculated based on the duration of time spent on exploring the novel object/the total duration of time spent on exploring the object and the novel object.

### Object-location memory task

The object-location memory task was conducted similarly to the novel object recognition test. Briefly, one of the walls displayed a horizontal stripes pattern, and two objects were placed in the corner, 6 cm away from the pattern. After the familiarization session (10 min), one of the objects was shifted to another corner that was 6 cm from the walls. The test session was conducted for 10 min, and the duration of time spent on the object's outer contour was calculated, as in the novel object recognition test.

### Social interaction test

The social interaction test was performed in an acrylic rectangular box (60 × 40 cm) that was divided into three compartments of equal size using retractable doors. The social interaction test was conducted over three sessions. Before the sociability test, the experimental mice were placed in the box for 5 min to acclimatize them to the new apparatus containing empty wire cages. At the end of the habituation session, each mouse was gently guided back to the central chamber, and the retractable doors were blocked. In the sociability session, a wire cage containing an encounter mouse (social zone) and an empty cage (neutral zone) were placed in one of the two side compartments. The test mouse was allowed to explore the three chambers for 5 min. Subsequently, the mouse was guided back to the middle compartment, and the retractable doors were blocked again. During the social novelty session, the empty cages were replaced with a new wire cage containing a novel mouse that the test mouse had never met before (novel zone), and the other cage was not disturbed (familiar zone). The test mouse was allowed to explore the chamber again for 5 min. The interaction time between the novel mouse and the empty cage was calculated using the EthoVision XT16, which tracked the nose-point of the test mouse and analyzed the total time spent in the cage's outer contour (2 cm). The social preference index was calculated as follows: (Social preference index) = (the duration of exploring the novel mouse)/(the total duration of exploring the empty cage (or familiar mouse) and the novel mouse).

### Rotarod test

The rotarod test was conducted for 2 consecutive days to evaluate both motor coordination and motor skill learning in mice. Rotarod was obtained from Panlab Harvard Apparatus (Barcelona, Spain). The mice were placed on a stationary rod for 60 s on the first day of the test. After habituation, three trials were conducted with 20-min inter-trial intervals (ITIs). During the trials, the rotation speed of the rod was accelerated from 4 to 40 rpm for a duration of 5 min. If the mouse fell off the rod within 10 s, the trial was repeated after 10 min. The latency to fall was recorded by the rotarod system when the test mouse fell off and touched the floor.

### Auditory fear conditioning test

The fear conditioning system (Panlab Harvard Apparatus, Barcelona, Spain) consisted of four chambers (25 × 25 × 25 cm^3^) that were placed in a sound-attenuating box (67 × 53 × 55 cm^3^). Auditory fear conditioning, extinction learning, and tone tests were performed under similar conditions (black wall and metallic grid floor), and each session was conducted one time daily. To pair the conditioned stimulus (CS; 2.8 kHz, 85 dB, 30 s) with an unconditioned stimulus (US, footshock; 0.3 mA, 0.5 s), experimental mice were acclimated in a chamber for 5 min, and an auditory CS was co-terminated with the US, which was delivered four times with 90-s inter-trial intervals. Extinction training was performed for the next 2 days. Mice were allowed to explore the chamber for 5 min, and then, an auditory CS was induced without the US. Extinction training was delivered 30 times per day. On the tone test day, the mice were allowed to explore for 5 min, and then five rounds of auditory CS were presented to test the mice. Freezing behavior was defined as a movement that lasts for more than 1 s. The parameters used in the fear conditioning system were as follows: 50 Hz sampling rate, 16x gain, and a disabled breathing filter.

### Statistical analysis

The prepared experimental mice underwent all behavioral tests; however, data that could potentially cause statistical errors and affect the final outcome were excluded. The data were excluded if the mouse fell off the floor in the EPM test (Walf and Frye, 2007) or if the mouse climbed onto an object in the novel object test and the object-location task. GraphPad Prism 8 software (GraphPad Prism) was used to perform all statistical analyses. All datasets were tested for normality by the Shapiro–Wilk test. If datasets did not show normal distribution, the Kruskal–Wallis test with Dunn's *post-hoc* test was used for comparisons across multiple groups. For datasets with normal distribution, one-way ANOVA or two-way ANOVA with repeated measures in one factor (rmANOVA) was used for comparisons across multiple groups. Tukey's *post-hoc* test was used for multiple comparisons after ANOVA. Two-tailed paired *t*-tests were performed to determine the preference of choice between two objects and two wired cages. A *p*-value of < 0.05 was considered statistically significant. The absence of statistical significance was indicated as non-significant.

## Results

### Locomotion and anxiety-related behavior

**Figure 2:**
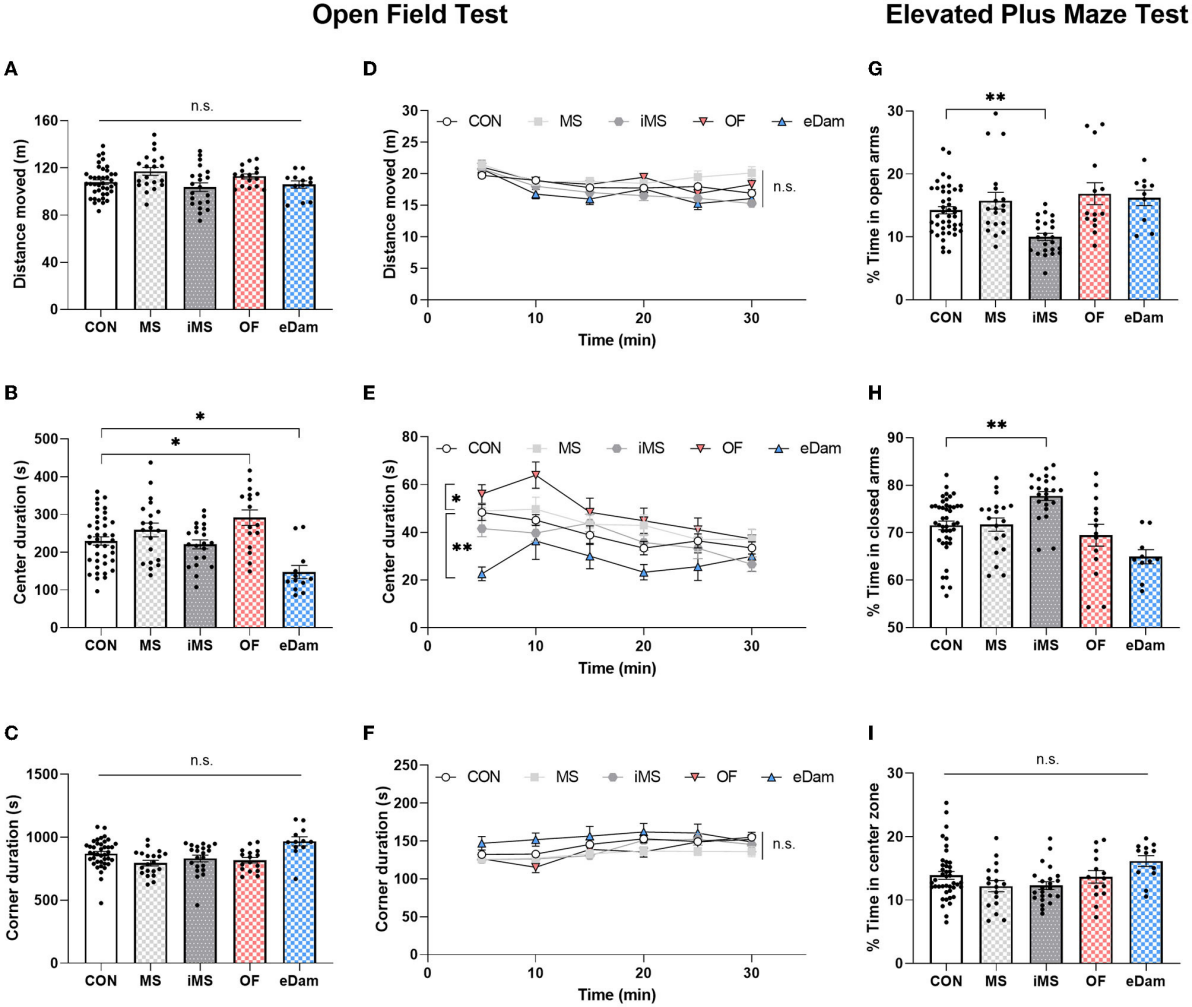
Maternal separation with the mother's odor enhanced the time spent at the center in the open field test; However, the dam-exchange group displayed reduced center duration. In addition, maternal separation in an empty room induced anxiety-related behavior. **(A)** Total distance moved for 30 min. **(B)** Time spent in the center area (CON, *n* = 39; MS, *n* = 20; iMS, *n* = 21; OF, *n* = 16; eDam, *n* = 12; *F*_(4,103)_ = 8.164, *p* < 0.0001, one-way ANOVA; CON vs. OF, *p* = 0.0295; CON vs. eDam, *p* = 0.0049; Tukey's *post-hoc* test). **(C)** Time spent in the corner area. **(D)** The total distance moved was analyzed in a 5-min time bin. **(E)** Time spent in the center was analyzed in a 5-min time bin [*F*_(4,106)_ = 5.252, *p* = 0.0007; two-way rmANOVA; CON vs. OF, *p* = 0.0029; CON vs. eDam, *p* = 0.0002; Tukey's *post-hoc* test]. **(F)** Time spent in the corner area was analyzed in a 5-min time bin. For the elevated plus maze test. **(G)** The percentage of time in open arms (CON, *n* = 44; MS, *n* = 19; iMS, *n* = 23; OF, *n* = 14; eDam, *n* = 10; *p* < 0.0001, Kruskal–Wallis test; CON vs. iMS: *p* = 0.0009, Dunn's *post-hoc* test). **(H)** Percentage of time in closed arms (*p* < 0.0001, Kruskal–Wallis test; CON vs. iMS: *p* = 0.001, Dunn's *post-hoc* test). **(I)** Percentage of time in the center zone. Circles represent data from individual animals, and bar graphs indicate mean ± SEM. n.s., not significant, *p < 0.05, **p < 0.001.

To investigate the impact of neonatal maternal separation under different environmental conditions on locomotor behavior, we conducted an open field test for 30 min when the mice reached PND 35. We measured the distance moved by each mouse and found that neonatal maternal separation did not affect the locomotor activity of the offspring (Figures 2A, D). However, we observed that the center duration was increased in litters in the OF group and reduced in those in the eDam group (Figures 2B, E). Additionally, all experimental groups showed no significant differences in corner duration during the open field test (Figures 2C, F). Behavioral paradigms of the animals in the open field test were interpreted in terms of locomotor activity, anxiety-related behavior, novelty-seeking behavior, and risk-taking behavior, and we determined that the change in center duration was likely due to the alteration in exploratory behavior, novelty-seeking behavior, in particular, and not anxiety-related behavior (Kiss et al., 2007; Wingo et al., 2016). Thus, we determined whether anxiety-related behavior affects the center duration in the open field test. We further examined anxiety-related behavior in detail using the elevated plus maze (EPM) test, which was found to be more reliable than the open-field test. As shown in Figure 2G, the time spent by the OF and eDam groups in the open arms was not different from the time spent by the CON group, suggesting that the change in center duration was due to the alteration in exploratory behavior, particularly the novelty-seeking behavior and not anxiety-related behavior. In contrast, the iMS group spent less time in the open arms (Figure 2G) and more time in the closed arms (Figure 2H). Additionally, all experimental groups exhibited no significant differences in the time spent in the center during the elevated plus maze (EPM) test (Figure 2I). Therefore, the results indicated that environmental conditions during neonatal maternal separation led to a change in adolescent behaviors and that their behavioral impact depends on various factors.

### Object recognition memory, place memory, and spatial working memory

**Figure 3:**
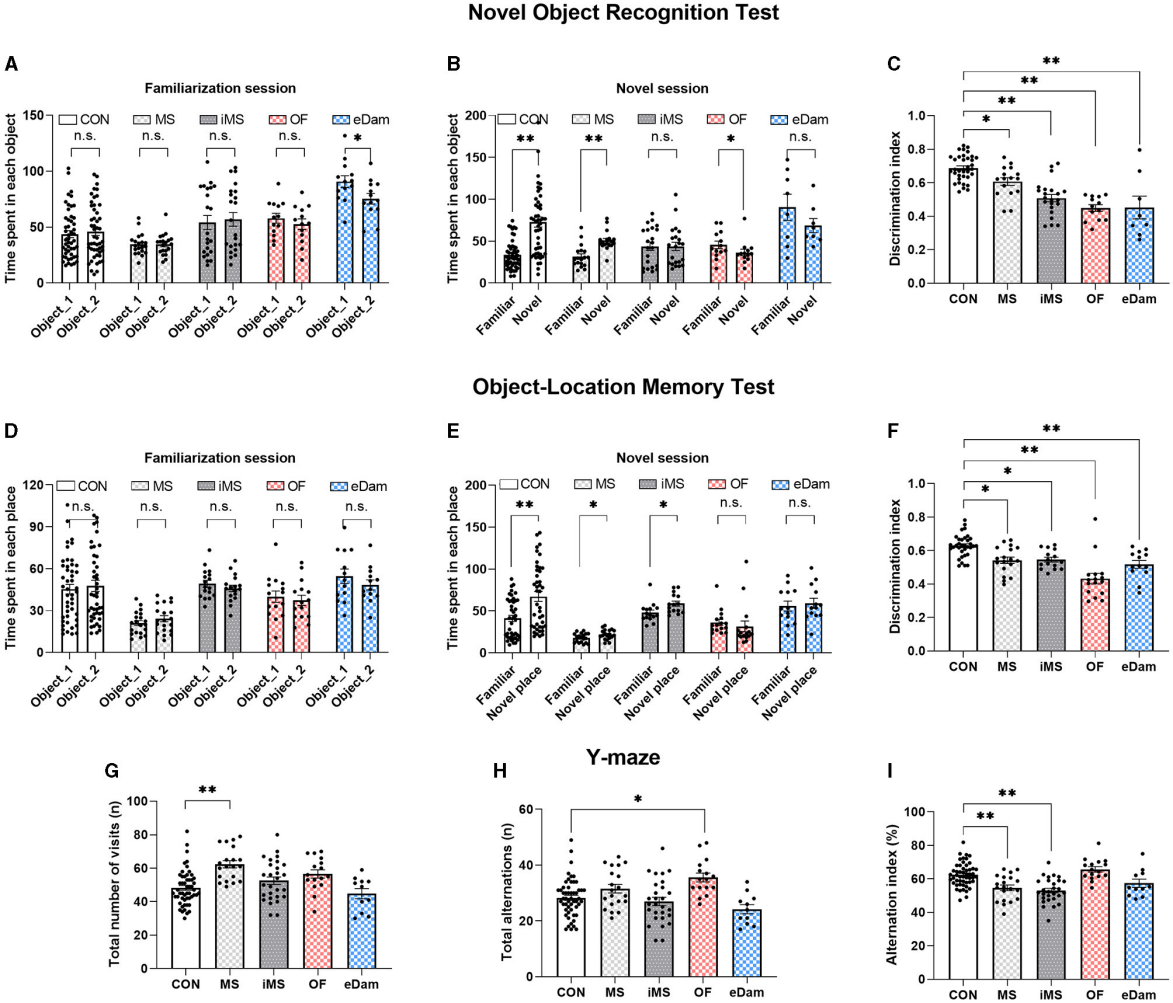
Maternal separation induced the impairment of recognition and memory of objects and places regardless of conditions. However, maternal odor or the existence of another dam ameliorated the impairment of spatial working memory. **(A)** Time spent exploring each object during the familiarization session (paired *t*-test, two-tailed; eDam, *p* = 0.021) **(B)** Time spent exploring each object during the novel session (CON, *n* = 49; MS, *n* = 18; iMS, *n* = 22; OF, *n* = 13; eDam, *n* = 8; paired *t*-test, two-tailed; CON, *p* < 0.0001; MS, *p* = 0.0002; OF, *p* = 0.0327). **(C)** Discrimination index for novel object test [*F*_(4,89)_ = 22.39, *p* < 0.0001, one-way ANOVA; CON vs. MS, *p* = 0.0331; CON vs. iMS, *p* < 0.0001; CON vs. OF, *p* < 0.0001; CON vs. eDam, *p* < 0.0001, Tukey's *post-hoc* test). **(D)** Time spent exploring each place during the familiarization session. **(E)** Time spent exploring each place during the novel session (CON, *n* = 42; MS, *n* = 19; iMS, *n* = 15; OF, *n* = 16; eDam, *n* = 13; paired *t*-test, two-tailed; CON, *p* < 0.0001; MS, *p* = 0.0263; iMS, *p* = 0.012). **(F)** Discrimination index for novel place test. [*F*_(4,91)_ = 16.31, *p* < 0.0001, one-way ANOVA; CON vs. MS, *p* = 0.0048; CON vs. iMS, *p* = 0.0147; CON vs. OF, *p* < 0.0001; CON vs. eDam, *p* = 0.0008, Tukey's *post-hoc* test). **(G)** The total number of arm entries [CON, *n* = 53; MS, *n* = 20; iMS, *n* = 28; OF, *n* = 16; eDam, *n* = 11; *F*_(4,123)_ = 8.476, *p* < 0.0001, one-way ANOVA; CON vs. MS, *p* < 0.0001, Tukey's *post-hoc* test]. **(H)** The number of spontaneous alternations [*F*_(4,123)_ = 6.361, *p* = 0.0001, one-way ANOVA; CON vs. OF, *p* = 0.003, Tukey's *post-hoc* test). **(I)** Alternation index [*F*_(4,123)_ = 13.06, *p* < 0.0001, one-way ANOVA; CON vs. MS, *p* = 0.0007; CON vs. iMS, *p* < 0.0001; MS vs. OF, *p* = 0.0001, Tukey's post-hoc test]. Circles represent data from individual animals, and bar graphs indicate mean ± SEM. n.s., not significant, *p < 0.05, **p < 0.001.

To investigate the effects of neonatal maternal separation under diverse conditions on cognition and recognition memory, we conducted a novel object recognition test and an object-location memory task. In the novel object recognition test, we observed that litters in the CON and MS groups displayed more interaction with the novel object than those with the familiar object (Figures 3A, B). Meanwhile, litters in the OF group showed a preference for the familiar object over the novel one. Additionally, in the object-location memory task, the CON, MS, and iMS group litters demonstrated a significant difference between the familiar and novel locations during the test session (Figures 3D, E). Although the MS and iMS group litters showed more interest in the novel stimulus, the discrimination index revealed a decrease in recognition memory in these groups than that observed in the CON group (Figures 3C, F). In both experiments, with the exception of the eDam group, there were no significant differences in the interaction time with the two objects during the familiarization session among the experimental groups (Figures 3A, D). The eDam group exhibited a tendency to interact more with one of the objects; however, no statistically significant differences were observed during the novel session (Figures 3A, B). Since these tasks tested the long-term memory of the mice (4 h), we examined short-term working memory using the Y-maze test. The MS and iMS groups scored a decreased alternation index (Figure 3I) although OF group showed a higher number of total alternations (Figure 3H) and the MS group showed an increased number of visits (Figure 3G). However, there were no significant differences in the alternation index compared to the CON group (Figure 3I). These results indicated that neonatal maternal separation under diverse conditions can impair recognition memory; however, the presence of maternal odor ameliorated the spatial working memory (Figure 3I).

### Social recognition memory and motor skill learning

**Figure 4:**
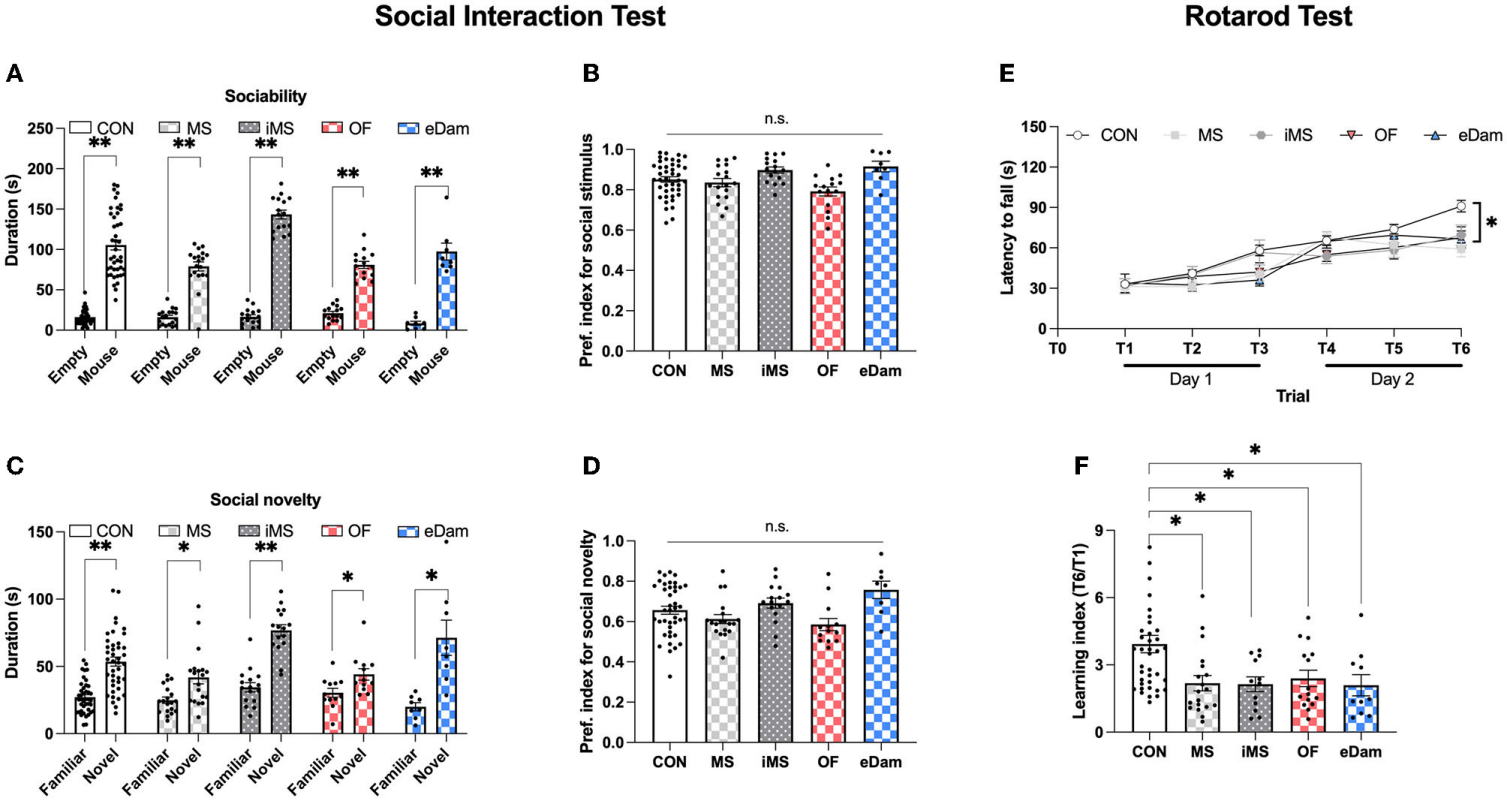
Maternal separation affected motor skill learning but did not affect sociality and social memory. **(A)** The time spent around wire cages in each place (CON, *n* = 42; MS, *n* = 19; iMS, *n* = 16; OF, *n* = 15; eDam, *n* = 8; paired *t*-test, two-tailed; CON, *p* < 0.0001; MS, *p* < 0.0001; iMS, *p* < 0.0001; OF, *p* < 0.0001; eDam, *p* = 0.0001) (Empty and Mouse represent the neutral zone and the social zone, respectively). **(B)** Preference index for sociability test. **(C)** The time spent around wire cages in each place (Paired *t*-test, two-tailed; CON, *p* < 0.0001; MS, *p* = 0.002; iMS, *p* < 0.0001; OF, *p* = 0.0157; eDam, *p* = 0.0052). **(D)** Preference index for the social novelty test (Familiar and Novel represent familiar zone and novel zone, respectively). **(E)** The latency to fall off the rotarod [CON, *n* = 44; MS, *n* = 20; iMS, *n* = 16; OF, *n* = 16; eDam, *n* = 14; *F*_(4,105)_ = 3.767, *p* = 0.0066, two-way rmANOVA; CON vs. MS, *p* = 0.0009; CON vs. iMS, *p* = 0.0485; CON vs. OF, *p* = 0.0062; CON vs. eDam, *p* = 0.0278, Tukey's *post-hoc* test]. **(F)** The learning index (T6/T1) (*p* = 0.0008, Kruskal–Wallis test; CON vs. MS, *p* = 0.0027; CON vs. iMS, *p* = 0.0287; CON vs. OF, *p* = 0.0467; CON vs. eDam, *p* = 0.0184, Dunn's *post-hoc* test). Circles represent data from individual animals, and bar graphs indicate mean ± SEM. n.s., not significant, *p < 0.05, **p < 0.001.

To further evaluate memory-related behavioral tests, we investigated social behavior and motor skill learning. In the social interaction test, litters in all experimental groups spent more time in the social zone than in the neutral zone (Figures 4A, B) and spent more time in the novel zone than in the familiar zone (Figures 4C, D). There was no significant difference in the social preference index among all five groups (Figures 4B, D). These results indicate that neonatal maternal separation did not affect sociality or social memory. We then assessed motor skill learning in the five groups using the rotarod test, which included six trials conducted for 2 consecutive days. While all five groups showed a gradual increase in the latency to fall, the latency to fall at T6 (day 2) was significantly reduced in the four maternal separation groups (MS, iMS, OF, and eDAM) when compared to the control group (CON) (Figure 4E). Furthermore, we calculated the learning index, which is the ratio of latency to fall during the sixth trial to latency to fall during the first trial, and confirmed that neonatal maternal separation impaired motor skill learning in the rotarod test (Figure 4F). These results were consistent with the findings of the memory tests. Thus, we can conclude that neonatal maternal separation under diverse conditions impaired memory in terms of recognition and motor skill learning.

### Auditory fear memory

**Figure 5:**
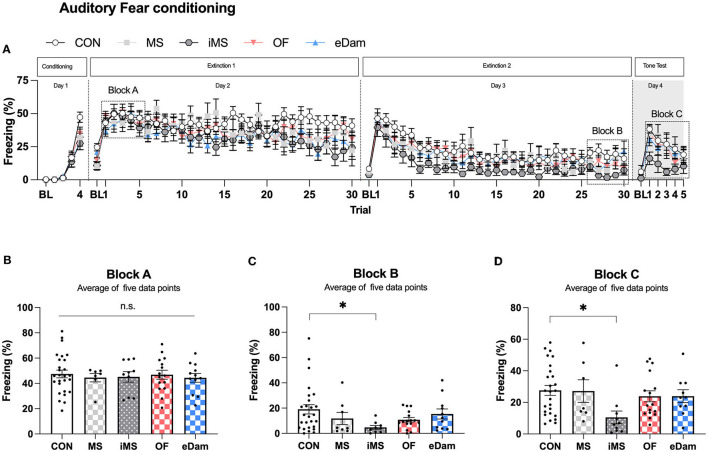
Maternal separation in an empty room provokes the enhancement of the extinction memory in the adolescent period. **(A)** Percentage of freezing behavior across the conditioning, extinction, and tone test sessions. Each circle represents the level of freezing when the conditioned stimulus (CS) was presented, except the first circle of each session, which shows the pre-CS baseline. **(B)** The average value of trials (1–5) on extinction day 1, which represents the extent of conditioning 1 day before extinction training. **(C)** The average value of trials (26–30) on extinction day 2 (CON, *n* = 26; MS, *n* = 8; iMS, *n* = 10; OF, *n* = 16; eDam, *n* = 12; *p* = 0.0778, Kruskal–Wallis test; CON vs. iMS, *p* = 0.0243, Dunn's *post-hoc* test). **(D)** The average value of trials (1–5) on tone test (*p* = 0.0202, Kruskal–Wallis test; CON vs. iMS, *p* = 0.0042, Dunn's *post-hoc* test). Circles represent data from individual animals, and bar graphs indicate mean ± SEM. n.s., not significant, *p < 0.05.

We investigated the effects of maternal separation on auditory fear memory, a long-lasting type of associative memory that is highly stable and durable. In this study, we presented four pairings of the CS and US to induce fear memory. After fear conditioning, we conducted extinction training for 2 days and then examined the freezing level of the CS on the day after the last extinction training (Figure 5A). We hypothesized that the acquisition of fear memory could be affected by different conditions since neonatal maternal separation had previously been shown to impair memory. However, we found no significant difference in the acquisition of auditory fear memory between the groups (Figure 5B). Extinction training was conducted for 2 days after fear conditioning, and we observed that the iMS group showed lower freezing levels on the second day of extinction training when compared to the other experimental groups (Figure 5C). This finding was confirmed by testing the fear response of mice to the CS on day 4 after extinction training (Figure 5D). Thus, we found that the mice placed in an isolated room during neonatal maternal separation had significantly enhanced extinction of fear memory, but no effect was noticed on the acquisition or retention of fear memory.

## Discussion

Our study found that neonatal maternal separation can cause changes in adolescent behavior, particularly in memory-related behaviors like recognition memory and motor skill learning. Additionally, we observed changes in other behaviors that were affected by environmental factors, such as exploratory behavior, anxiety-related behavior, spatial working memory, and auditory fear memory. The introduction of maternal odor and other dams during the maternal separation improved spatial working memory but did not alleviate deficits in recognition memory. During the maternal separation procedure, completely isolated mice (iMS) exhibited the most significant change in behavior, with increased anxiety-related behavior, enhanced extinction memory, and deficits in recognition memory. Our findings provide evidence that exposure to different environmental conditions during neonatal maternal separation can lead to alterations in adolescent behaviors and that the impact of these changes depends on various factors.

Even though the open field test was useful in screening for emotional behaviors, it was also affected by various factors, such as social isolation, novel environments, and open spaces, which further affected general anxiety and exploratory behavior. Therefore, we interpreted the results using combined behavioral outcomes rather than focusing on the individual results of the open-field test. In the open field test, the OF group displayed an increased center duration, whereas the eDam group spent less time in the central area. Since the OF and eDam groups did not show differences in the EPM test when compared to the CON group, it is likely that olfactory stimulation during maternal separation might have modified the odor-evoked circuitry and enhanced the exploratory behavior to seek novelty. Indeed, the maternal odor is responsible for a strong correlation between the PFC-amygdala/hippocampus and the PFC-olfactory cortex at PND 14 and PND 23 (Perry et al., 2016). This change in circuitry also enhanced the behaviors related to the interaction between mother and infant, such as nipple attachment, threat, and learning to avoid predators (Moriceau et al., 2006; Raineki et al., 2010; Upton and Sullivan, 2010). Therefore, our results suggest that exposure to maternal odor during repeated maternal separation may lead to enhanced exploratory behaviors in adolescent mice.

Previously, the presence of maternal odor during separation had been reported to induce an abrupt increase in the heart rate, whereas the odor of other females or no odor did not affect the cardiac rate (Compton et al., 1977). This observation supports the notion that pups can recognize their dams and distinguish them from other females. The pups left with other dams (eDam) may notice differences between their dams and others. However, contrary to our expectations, mice in the eDam group exhibited unexpected behavioral changes in the open field test and exhibited reduced exploratory behavior than those of the CON group. We speculated that the presence of other dams may act as another stress factor and lead to decreased exploratory behavior.

Pups are deaf for the first 10 days after birth (Moore et al., 1983), and the rate of ultrasonic vocalization (USV) induced by maternal separation increases during the first week of pup life (Elwood and Keeling, 1982). The production of USV in mice is a complicated phenomenon. Pups produce many calls with a frequency above 100 kHz, whereas adult mice produce lower-frequency calls (Grimsley et al., 2011). Intriguingly, previous studies have suggested that environmental modulation can induce modifications in USV. Pups during maternal separation emit fewer USVs when exposed to nest odor or mother's odor than when exposed to clean bedding (D'amato and Cabib, 1987; Moles et al., 2004; Wohr, 2015). In addition, pups raised with additional female mice showed decreased USV emission during maternal separation (Oddi et al., 2015). Furthermore, the audience effect demonstrated that male vocal behavior during male–female mouse courtship can be altered in the presence of listeners (Seagraves et al., 2016). Our data, in line with previous studies, showed different behavioral impacts on the MS mice, those that moved to another vivarium, and the iMS mice, which was isolated in an empty room, indicating that social environment during the neonatal period is an important factor for developing anxiety-related behaviors and fear extinction memory in adolescents.

In the stress model, exposure to various types of stressors (both acute and chronic), such as maternal separation, predator exposure, social defeat, and social isolation, has been linked to deficits in fear extinction (Garcia et al., 2008; Wilber et al., 2009; Clay et al., 2011; Chauveau et al., 2012; Dubreucq et al., 2012; Wilson et al., 2013; Zhang and Rosenkranz, 2013; Xiong et al., 2014). It has also been reported that stress hormones, such as norepinephrine, can modulate the consolidated state of fear memory (Lee et al., 2011) and that stress can induce changes in α-amino-3-hydroxy-5-methyl-4-isoxazolepropionic acid (AMPA) receptor subunit phosphorylation in the amygdala, which subsequently affects the quality of fear extinction (Caudal et al., 2010; Lee et al., 2013). Extinction memory is known to vary significantly according to age. Infants typically exhibit an erasure-like form of extinction during their normal growth process, whereas adult mice show an extinction memory similar to the spontaneous recovery of extinction. The degree of impairment of extinction memory is higher in adolescent mice than in infant and adult mice (Kim and Richardson, 2007; Kim et al., 2009). Furthermore, the timing of stressor exposure is an important factor in the development of fear extinction memory. Exposure to stressors during infancy leads to the emergence of the adult form of extinction (Callaghan et al., 2013), indicating that the presence of stress early in life can facilitate the normal development of extinction memory. Thus, it is possible that repeated maternal separation in early life may promote the ontogeny of extinction. Studies have shown that stress models show enhanced extinction memory. For example, maternal separation has been found to improve the extinction of social fear without affecting social memory, sociality acquisition, or expression of social fear (Zoicas and Neumann, 2016). Additionally, placing rats on an elevated platform for 30 min facilitated extinction memory and extinction retrieval post-weaning (Schayek and Maroun, 2015). In line with these findings, neonatal maternal separation may induce alterations in the extinction circuitry, particularly in the medial prefrontal cortex (mPFC) and hippocampal neurons, which interact with the amygdala to regulate extinction (Izquierdo et al., 2006; Holmes and Wellman, 2009; Leuner and Gould, 2010). Interestingly, placing rats on an elevated platform disrupted mPFC-basolateral amygdala (BLA) plasticity and impaired extinction in some studies (Maroun and Richter-Levin, 2003; Rocher et al., 2004; Maroun, 2006; Richter-Levin and Maroun, 2010), while enhancing it in others (Schayek and Maroun, 2015), indicating that the effects of stress-induced neuronal alterations vary with age.

Our study found that anxiety-like behaviors increased when pups were separated from their dams and moved to another room, which is consistent with the findings of previous studies (Shin et al., 2016; Kim et al., 2023). Studies have also shown that an increase in ultrasonic vocalizations, which indicate anxiety-like behavior, occurs when maternal separation is performed in a different room (Kestering-Ferreira et al., 2021). However, anxiety-like behavior does not appear in cases where pups are not isolated in another room, such as by cross-fostering or transferring to a dam instead of pups (Luchetti et al., 2015; Jarrar et al., 2022). Our eDam group corresponds to the cross-fostering model, and the OF group corresponds to transferring the dam instead of the pups as the mother's scent remains in the cage. Therefore, the absence of anxiety-like behavior corresponds to the results of this study, which serves as an example to explain some discrepancies between studies. In contrast, neonatal maternal separation can lead to memory deficits, which manifest as a distinct phenotype. Our results are consistent with previous studies showing that memory deficits are a prominent symptom observed in strains subjected to maternal separation, such as BALB/c, C57BL/6, and other strains (Tractenberg et al., 2016). Memory deficits are considered to be a representative symptom of maternal separation and constitute the first criterion used to evaluate the effectiveness of maternal separation.

The present findings displayed that maternal separation may affect multiple regions of the brain and alter adolescent behavior. Previous studies have shown that maternal separation can cause changes in the substantia nigra (Rots et al., 1996), as well as in dopamine transporters and stress response to dopamine (Hall et al., 1999; Meaney et al., 2002). Although our study concluded that diverse maternal separation conditions can lead to deficits in motor skill learning based on a series of memory tests, we cannot exclude the possibility that the impact of maternal separation, as an early-life environmental stress factor, on the dopamine system may be correlated with the development of Parkinson's disease (He et al., 2020).

The results of our behavioral tests indicate that the hippocampus, mPFC, and paraventricular nucleus of the thalamus (PVT) are involved in the maternal separation paradigm (Bird and Burgess, 2008; Fiedler et al., 2021; Tao et al., 2021). The hippocampus has been extensively studied in the brain structure of the maternal separation paradigm (Fabricius et al., 2008; Zalosnik et al., 2014; Shin et al., 2016; De Azeredo et al., 2017; Maghami et al., 2018) to determine the relationship between memory performance and cellular (Hulshof et al., 2011; Bachiller et al., 2020) and genetic alterations (De Azeredo et al., 2017; Alberry et al., 2020). Additionally, two projections, from mPFC to PVT (Do-Monte et al., 2015; Tao et al., 2021) and from PVT to the central amygdala (Penzo et al., 2015), mediate the retrieval of fear extinction (Do-Monte et al., 2015; Tao et al., 2021). Therefore, our results suggest that the impact of early-life stress factors on brain regions is influenced by the environmental conditions during maternal separation. Further investigation is needed to determine the underlying mechanisms of individual phenotypes. However, our findings provide evidence that individual environmental interventions during the maternal separation process affect adolescent behaviors. Thus, it is essential to pay particular attention to constructing an appropriate stress model to achieve consistency in the future.

Finally, our study focused on the adolescent period to enhance the validity and reliability of the test. We did not examine the adult period to rule out unexpected environmental impacts during the duration of mouse breeding. Although we cannot be certain about how the effects of neonatal maternal separation change over time from adolescence to adulthood, several studies help us speculate how they may change. For example, exposure to juvenile stress in rats leads to long-term effects on synaptic changes in the hippocampus during adulthood (Raghuraman et al., 2022). Additionally, maternal separation (PND 1–21) has been found to cause long-term effects, including anxiety-like behavior and impaired recognition phenotype during adolescence, which persist into adulthood (Banqueri et al., 2017). However, in the same study, it was found that shorter periods of maternal separation (PND 1–10) only deteriorated associative/emotional learning during adolescence (Banqueri et al., 2017). Conversely, another study observed anxiety and depression symptoms during adolescence, but only depression symptoms persisted until adulthood (Chen et al., 2021). Therefore, it is possible that the behavioral changes we observed during adolescence may not persist into adulthood, with anxiety symptoms potentially resolving with time while memory deficits persist.

## Data availability statement

The original contributions presented in the study are included in the article/supplementary material, further inquiries can be directed to the corresponding author.

## Ethics statement

The animal study was reviewed and approved by the Institutional Animal Care and Use Committees of the Korea Brain Research Institute (KBRI).

## Author contributions

SL and SS conceptualized and designed the study and wrote the manuscript. SS performed behavioral assessment and data analysis. All authors contributed to the article and approved the submitted version.
